# Notch1 Affects Chemo-resistance Through Regulating Epithelial-Mesenchymal Transition (EMT) in Epithelial Ovarian cancer cells

**DOI:** 10.7150/ijms.44683

**Published:** 2020-05-18

**Authors:** Xue-qian Qian, Sang-sang Tang, Yuan-ming Shen, Li-li Chen, Xiao-dong Cheng, Xiao-yun Wan

**Affiliations:** Women's Reproductive Health Key Laboratory of Zhejiang Province; Department of Gynecologic Oncology, Women's Hospital, School of Medicine, Zhejiang University, and Hangzhou, China

**Keywords:** Notch1, EOC cells, EMT, ovarian cancer

## Abstract

**Background**: Epithelial ovarian cancer (EOC) is the most lethal gynecological malignancy, chemo-resistance is the main cause for treatment failure. Our previous studies have found that SKOV3 could promote immune escape and tumor progression via Notch1 pathway. Therefore, Notch1 is suspected to be involved in chemo-resistance. The current study is to investigate the possible mechanisms of platinum-resistance in epithelial ovarian cancer mediated by Notch1.

**Methods**: The expressions of Notch1, Snail, MMP-2, N-cadherin, Vimentin and E-cadherin were detected by Western-blot. A stable high expression or low expression of Notch1 in ovarian cancer cells was established by using lentiviral gene engineering. The cell migration and invasion ability were observed by scratch test and transwell test. Cell apoptosis rate and cell cycle were analyzed by flow cytometry.

**Results**: The expression levels of Notch1, Snail, MMP-2, N-cadherin and Vimentin in ovarian cancer were high, while the expression levels of E-cadherin were low.Notch1 promoted the expression of Snail, vimentin, N-cadherin and MMP2 protein, but inhibiting the expression of E-cadherin, promoting cell migration and invasion. Notch1 affected apoptosis of cells through Epithelial-Mesenchymal Transition (EMT), increasing the proportion of cells in S phase and G2 phase, thus affecting drug resistance.

**Conclusion**: Notch1 affects EOC cells chemo-resistance by regulating EMT. This may provide a new target for the treatment of ovarian cancer.

## Background

Epithelial ovarian cancer (EOC) is the most lethal gynecological malignancy. Although most patients experience clinical complete remission after operation combined with chemotherapy, the 5-year survival rate remains at 30-40%. For recurrent ovarian cancer, chemo-resistance is the main cause for treatment failure and current treatment strategies are limited. More and more studies have confirmed the existence of genetic and epigenetic abnormalities in EOC 1-3. Targeting these abnormal molecular and cellular signaling pathways is becoming a new treatment option for those patients.

SKOV3 is one of the most typical EOC cell lines, and studies revealed that MDM2 could promote epithelial and mesenchymal transition using SKOV3 4,23, and our previous studies also have found that SKOV3 could affect the differentiation and function of dendritic precursor cells via Notch1 pathway, promoting immune escape and tumor progression 5. Recently, it has been reported that Notch1 signaling pathway is involved in the development and drug resistance of ovarian cancer 6. Epithelial-mesenchymal transition (EMT) is a process by which epithelial cells lose their cell polarity and cell-cell adhesion, and gain migratory and invasive properties to become mesenchymal stem cells 7. Epithelial cells express high levels of E-cadherin, whereas mesenchymal cells express those of N-cadherin, fibronectin and vimentin. Loss of E-cadherin is considered to be a fundamental event in EMT. Snail can bind to the E-cadherin promoter and repress its transcription. Recent literature considered that EMT was associated with chemo-resistance. Zheng et al. reported that EMT was dispensable for metastasis but induced chemo-resistance in pancreatic cancer 8. Fischer KR et al. reported that EMT helped to resist chemotherapy 9. However, whether Notch1 regulates EMT to induce chemo-resistance ovarian cancer has not been reported.

Based on our previous research, this study is aimed to address the expression of Notch1 and EMT molecules in EOC. Results from this study will be used to illustrate the relationship between Notch1 and the EMT process, as well as confirm that Notch1 affects chemo-resistance via regulating EMT.

## Materials and Methods

### Patients and samples

All the samples were collected in Women's Hospital, School of Medicine, Zhejiang University between June 1, 2016 to December 30, 2016. The collection of all samples was approved by the Ethical Committee of the hospital. All the tissue samples were confirmed by pathological examination and were divided into three groups: (i): ovarian cancer tissue; (ii): opposite normal ovary tissue matched with unilateral ovarian cancer (paired control); (iii): normal ovarian tissue from benign disease (normal control).There were 5 cases in each group. The proteins were obtained by grinding the tissue and adding the protein lysate. Western blot was used to determine the protein expression of Notch1 and EMT related molecules. The clinical characteristics of each group were consistent, including age, pathology, etc. Five samples in each group were mixed together according to reported literature 10.

### Cell culture

An epithelial ovarian carcinoma cell line SKOV3 was purchased from American Type Culture Collection. SKOV3 was prepared in 1640 culture medium containing 10% fetal bovine serum (FBS) and 1% double antibody (mixed with penicillin streptomycin) at 37 °C. The culture was carried out in an incubator of 5% CO2. The cells were collected for future use.

### Lentivirus construction and transfection

To generate Notch intracellular domain (NICD) and Snail over-expression or low-expression stable transfect ants, EOC cells were transfected with lentiviral expressing vectors. The plasmid construction and lentivirus package were completed in GENECHEM Company. The SKOV3 cells were seeded at 5×10^4^ cells/well into 96-well plates at 24-hour prior to transfection. When the cells were fused to 60-70%, lentivirus particles were transfected using the manufacturer's protocol.

### Western Blot Analysis

The primary antibodies used were anti- Notch1(1:500), anti-E-cadherin (1:2000), anti-vimentin (1:4000), anti-N-cadherin (1:500), anti-MMP-2(1:500). anti-Snail (1:1000), anti-Caspase3 (1:600), anti-Bcl2 (1:1000), anti-Bax (1:2000) and anti-GAPDH (1:2000) as an endogenous control, all from Epitomics Biotechnology (Epitomics). HRP-conjugated seconddary antibodies (goat anti-rabbit IgG or goat anti-mouse IgG, 1:2000) were from Cell Signaling (Beverly, MA).Moreover, ECL (Enhanced Chemical Luminescence) and gel imager were used for imaging.

### Wound Healing Assay

Wound healing assay was carried out to evaluate migration ability of tumor cells. At 5 H post-transfection in a six-well plate on SKOV3 cells, scratch was made in length and breadth through the center of each well using a pipette tip, an open wound was created without cells. Cells migrated into the wounded area. A photograph was taken at 0, 24, 48, 72, 96, 120H post-scratching.

### Cell Cycle Analysis

The cells in the logarithmic growth stage were digested by trypsin and were inserted into the six orifice plates with the density of 3 x 10^5^ cells/holes. When the cells grew to 60-70% mixture, transfection was carried out. Cells were treated with cisplatin (80 g/ml) before the cells were harvested for 24 hours. After 48 H post-transfection, the cells were washed with cold PBS and fixed with 70% alcohol for 4 H. Then, the cells were stained with propidium iodide (50μg/mL) for 10 Min at 37 ℃, in the dark. Flow cytometry (Beckman Coulter Epics) was used to calculate the percentage of cells in G0/G1, S, and G2/M phases with FLOWJO software.

### Cell Apoptosis Analysis

After 48 H post-transfection, apoptotic cells were stained using Annexin-V/PI according to the manufacture's protocol. Flow cytometry was used to evaluate cell apoptosis rate.

### Cell Invasion Analysis

Invasion assay was measured using Biocoat Matrigel invasion chambers (BD Biosciences). At 24 H post-transfection, SKOV3 cells were imported into the upper compartment of the Transwell. Matrigel was added to each well in the transwell plate, then incubated to solidify at 37℃. The percentage invasion was calculated using a microscope as the number of cells in six randomly selected fields per well. The experiment was repeated three times.

### Immunofluorescence staining

SKOV3 cell proliferation was analyzed by ki67 and 5-ethynyl-2′-deoxyuridine (Edu) immunofluorescence staining. The primary antibody used was anti- Ki67 (1:50, Abcam,USA) and secondary antibody used was goat anti-rabbit IgG(1:400, Jackson, USA). Edu (RiboBio, Germany) solution was diluted with cell culture medium at a ratio of 1000: 1, 50 μM of Edu was added to the media and the material was incubated for two hours. Detection was performed with the Leica biological microscope (Germany) as described by the manufacturer for sections.

### Statistical Analysis

Every experiment was independent and repeated at least three times. All the figure we provided represented only one of the multiple experiments. The statistical significance was determined using Student's t-test analysis using Prism 5 software (GraphPad). Data were considered to be statistically significant when *P < 0.05, **P < 0.01, ***P < 0.001.

## Results

### Western blot detecting the expression of Notch1 and EMT related molecules

**Figure 1:**
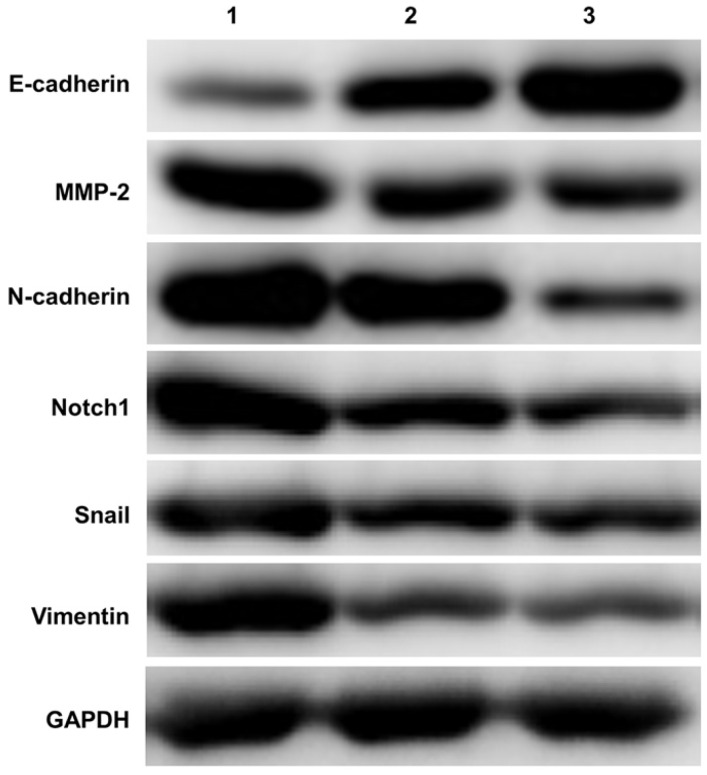
** Western blot detecting the expression of Notch1 and EMT related molecules.** Ovarian cancer tissue had increased expression of Notch1, Snail, MMP-2, N-cadherin and Vimentin, but had decreased expression of E-cadherin. (1 = Ovarian cancer tissue, 2 = Opposite normal ovarian tissue, 3 = Normal ovarian tissue)

A total of 15 ovarian tissue samples were collected to evaluate Notch1 and EMT related molecules expression by western-blot, including 5 of EOC with stage I (Cancer tissue was considered as group 1, Opposite normal ovarian tissue was considered as group 2), another 5 normal ovarian tissue from benign cyst were considered as group 3. As shown in Figure 1, Ovarian cancer tissue had increased expression of Notch1, Snail, MMP-2, N-cadherin and Vimentin, but had decreased expression of E-cadherin.

### Notch1 regulated the EMT process

#### The experimental groups

To confirm that Notch1 regulates EMT processes at the SKOV3 cellular level, we divided the experiment into the following 4 groups: (1) group 1 = pCDNA3.1(+), which represents eukaryotic expression vector; (2) group 2 = pCDNA3.1(+)-NICD, which indicates that the Notch1 intracellular segment (NICD) was constructed into the eukaryotic expression vector; (3) group 3 = Notch1 ctr siRNA, which represents blank interference; (4) group 4 = Notch1 siRNA, which represents Notch1 interference.

#### The evaluation of Notch1 and EMT related molecules expression in SKOV3 cell by Notch1 interference and transfection

**Figure 2:**
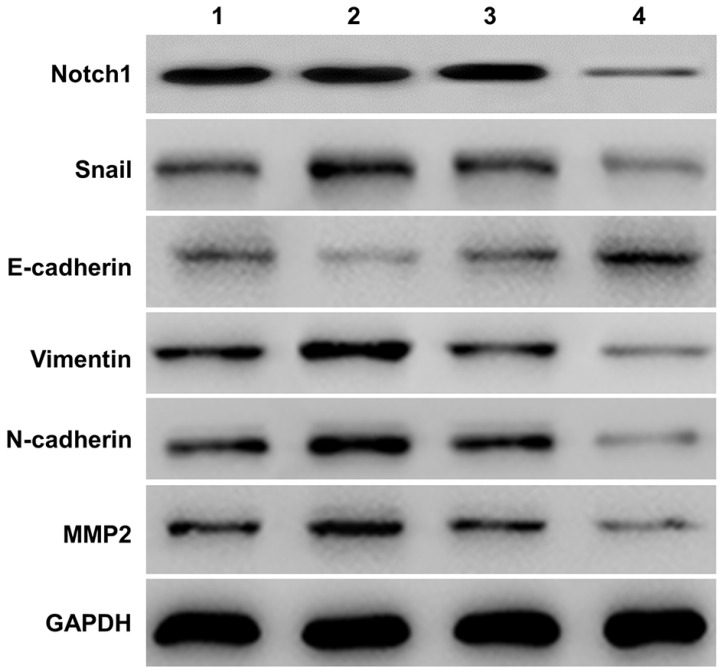
** Western-blot measured the expression of E-cadherin and other EMT related proteins (Vimentin, N-cadherin, and MMP2).** Compared with the NICD over-expression group (group 2 vs group 1), the expression of Notch1, Snail, Vimentin, N-cadherin, MMP2 protein increased, and the expression of E-cadherin decreased. The expression of E-cadherin and other EMT related proteins was totally opposite in cells infected with Notch1-siRNA (group 4 vs group 3). (1 = pCDNA3.1(+), 2 = pCDNA3.1(+)-NICD, 3 = Notch1 ctr siRNA, 4 = Notch1 siRNA)

Western-blot measured the expression of E-cadherin, Vimentin, N-cadherin and MMP2) in SKOV3 cells (See Figure 2). Compared with the NICD over-expression group (group 2 vs group 1), the expression of Notch1, Snail, Vimentin, N-cadherin, MMP2 protein increased, and the expression of E-cadherin decreased. However, siRNA mediated Notch 1 knock-down significantly increased the expression of E-cadherin but reduced the expression of vimentin, N-cadherin, and MMP2 (group 4 vs group 3).

#### The evaluation of migration and invasion capacity in SKOV3 by Notch1 interference and transfection

**Figure 3:**
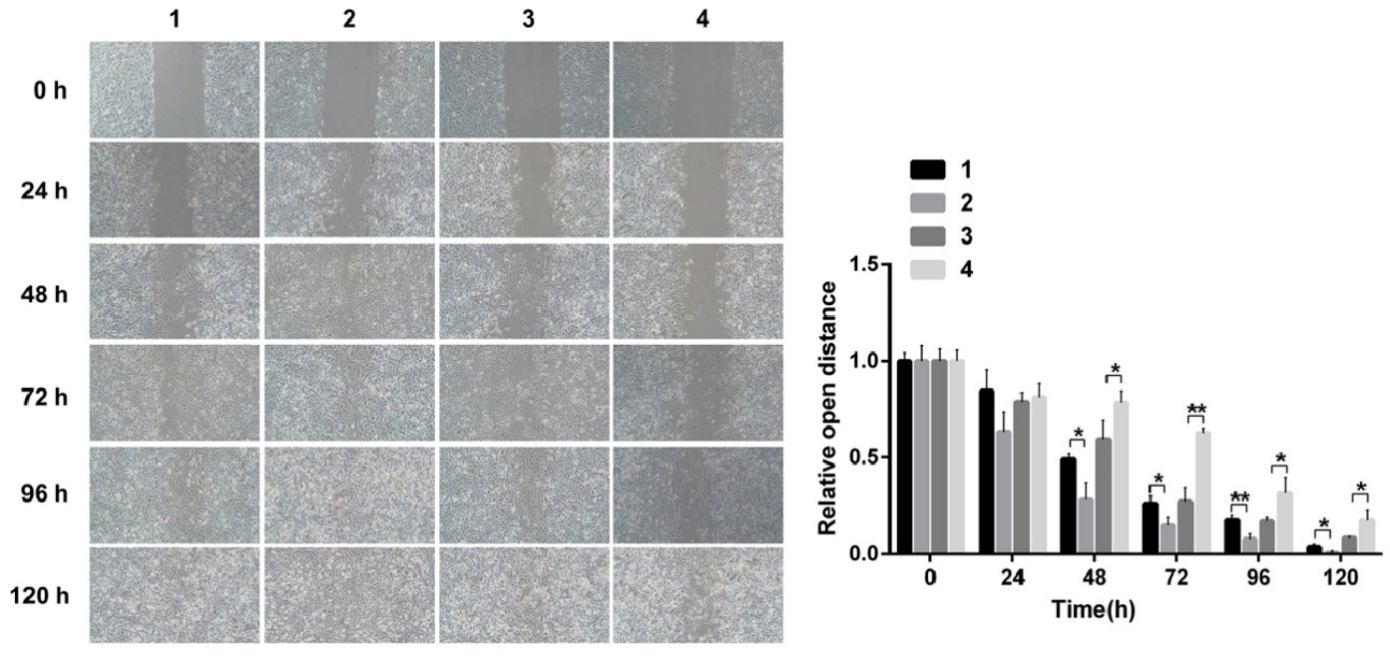
** Scarification test.** The results showed the cell migration capacity increased in cells infected with pCDNA3.1(+)-NICD and decreased in the cells infected with Notch1-siRNA at 24 h, 48 h, 72 h, 96h and 120h, respectively. (1 = pCDNA3.1(+), 2 = pCDNA3.1(+)-NICD, 3 = Notch1 ctr siRNA, 4 = Notch1 siRNA)

Scarification tests showed the cell migration capacity increased in cells infected with pCDNA3.1(+)-NICD and decreased in the cells infected with Notch1-siRNA at 24 h, 48 h, 72 h, 96h and 120h, respectively. (Figure 3)

**Figure 4:**
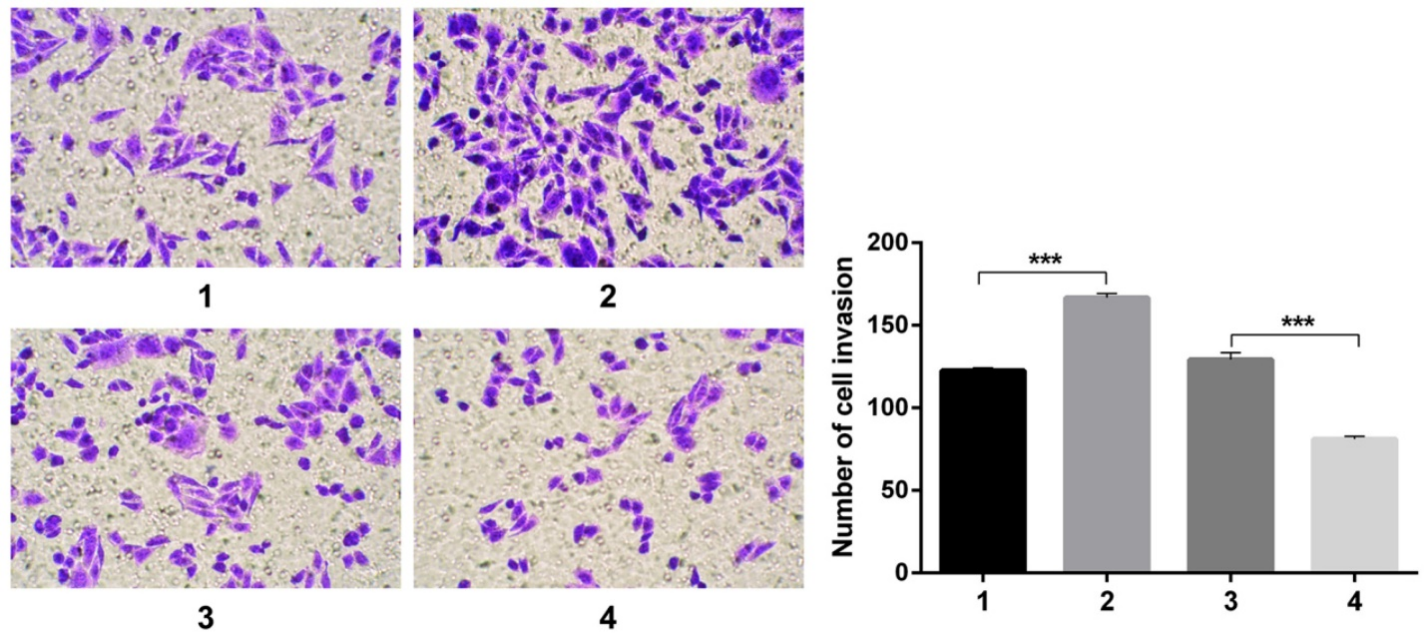
** Transwell experiment.** The results revealed an increase in cell invasion infected with pCDNA3.1(+)-NICD and a decrease in cell invasion infected with Notch1-siRNA. (1 = pCDNA3.1(+), 2 = pCDNA3.1(+)-NICD, 3 = Notch1 ctr siRNA, 4 = Notch1 siRNA)

Transwell experiments revealed an increase in cell invasion infected with pCDNA3.1(+)-NICD and a decrease in cell invasion infected with Notch1-siRNA. All the figure we provided represented only one of the multiple experiments (Figure 4) (*P≤0.05, **P≤0.01, ***P < 0.001.).

### Notch1mediated acquired chemo-resistance via facilitating EMT

#### The experimental groups

To confirm that Notch1mediated acquired chemo-resistance via facilitating EMT at the SKOV3 cellular level, we divided the experiment into the following 6 groups: (1) group 1 = Snail NC + pCDNA3.1(+) + Cisplatin; (2) group 2 = Snail NC + pCDNA3.1(+)-NICD + Cisplatin; (3) group 3 = Snail siRNA+pCDNA3.1(+)-NICD + Cisplatin; (4) group 4 = pCDNA3.1(+) + Notch1 NC + Cisplatin; (5) group 5 = pCDNA3.1(+) + Notch1 siRNA + Cisplatin; (6) group 6 = pCDNA3.1(+)-Snail + Notch1 siRNA+ Cisplatin.

#### Notch1 and EMT related proteins expression change in SKOV3 cells after Snail inducement

**Figure 5:**
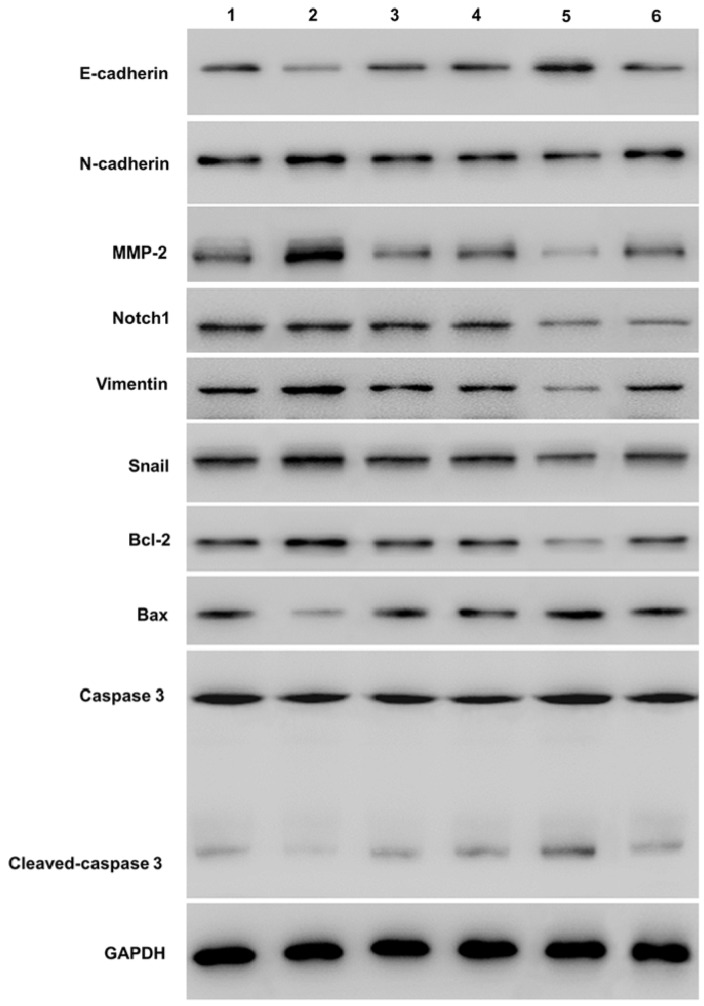
** Western blot measuring Notch1 and EMT related proteins expression after Snail interference.** Western-blot showed that over-expression of Notch1 decreasing E-cadherin, caspase3, cleaved-caspase3, and increasing vimentin, N-cadherin, MMP2 and Bcl2 (group 1 vs group 2). However, siRNA mediated Notch 1 knock-down had the opposite effect (group 4 vs group 5). Snail would reverse the effect (group 2 vs group 3; group 5 vs group 6). (1 = Snail NC+pCDNA3.1(+)+Cisplatin, 2 = Snail NC + pCDNA3.1(+)-NICD + Cisplatin, 3=Snail siRNA+pCDNA3.1(+)-NICD+Cisplatin; 4= pCDNA3.1(+)+Notch1 NC Cisplatin; 5 = pCDNA3.1(+) + Notch1 siRNA + Cisplatin; 6 = pCDNA3.1(+)-Snail + Notch1 siRNA + Cisplatin)

Western-blot showed that over-expression of Notch1 decreasing E-cadherin, caspase3, cleaved-caspase3, and increasing vimentin, N-cadherin, MMP2 and Bcl2 (group 1 vs group 2, P < 0.001). However, siRNA mediated Notch 1 knock-down had the opposite effect (group 4 vs group 5, P < 0.001). Snail would reverse the effect (group 2 vs group 3, p<0.001; group 5 vs group 6, P < 0.001). (*P≤0.05, **P≤0.01, ***P < 0.001.) (Figure 5).

#### Flow cytology analysis of cell cycle and apoptosis rate after Snail inducement

**Figure 6:**
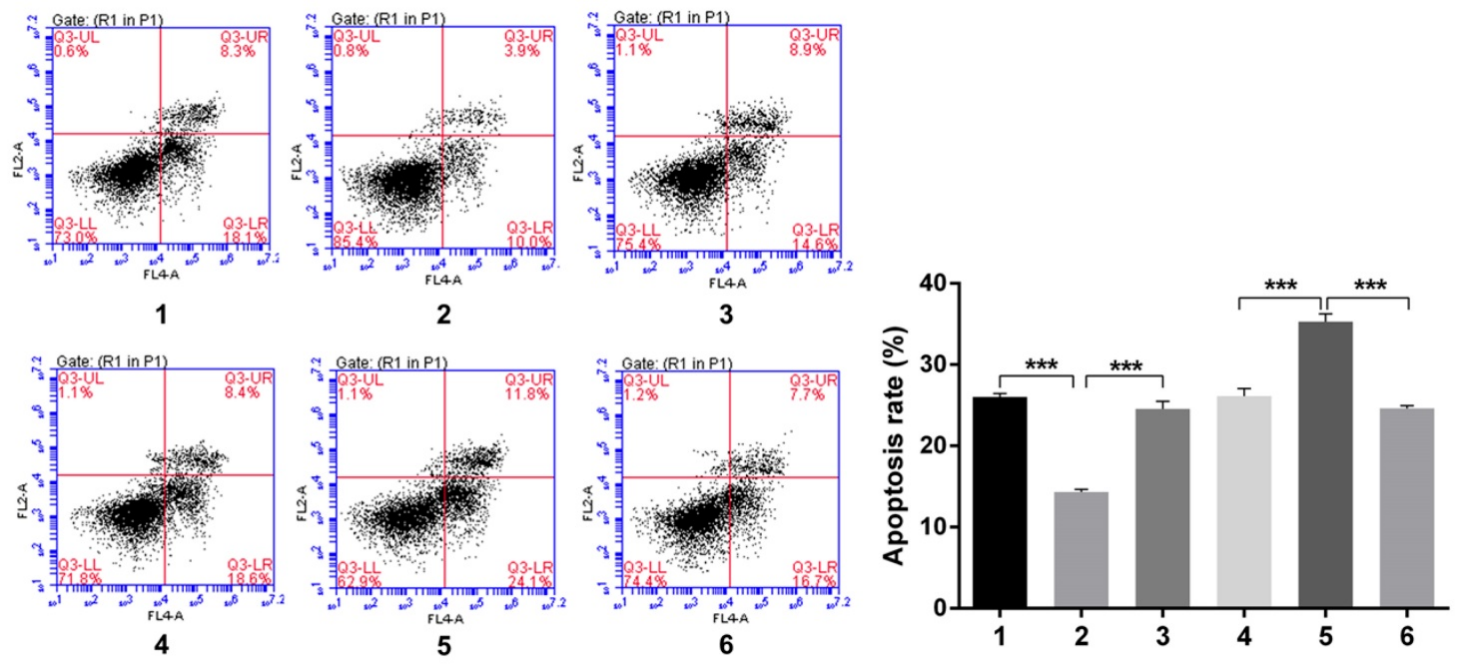
** Flow cytology analysis of apoptosis rate after Snail interference.**Over-expression of Notch1 inhibited the apoptosis rate (group 1 vs group 2), while siRNA mediated Notch1 knock-down promoted apoptosis (group 4 vs group 5), Snail would reverse the effect (group 2 vs group 3; group 5 vs group 6). (1 = Snail NC + pCDNA3.1(+) + Cisplatin, 2 = Snail NC + pCDNA3.1(+)-NICD + Cisplatin, 3 = Snail siRNA+pCDNA3.1(+)-NICD + Cisplatin; 4 = pCDNA3.1(+) + Notch1 NC + Cisplatin; 5 = pCDNA3.1(+) + Notch1 siRNA + Cisplatin; 6 = pCDNA3.1(+)-Snail + Notch1 siRNA+ Cisplatin)

Over-expression of Notch1 inhibited the apoptosis rate (group 1 vs group 2, p<0.001), while siRNA mediated Notch1 knock-down promoted apoptosis (group 4 vs group 5, p<0.001), Snail would reverse the effect (group 2 vs group 3, p<0.001; group 5 vs group 6, p<0.001) (Figure 6).

**Figure 7:**
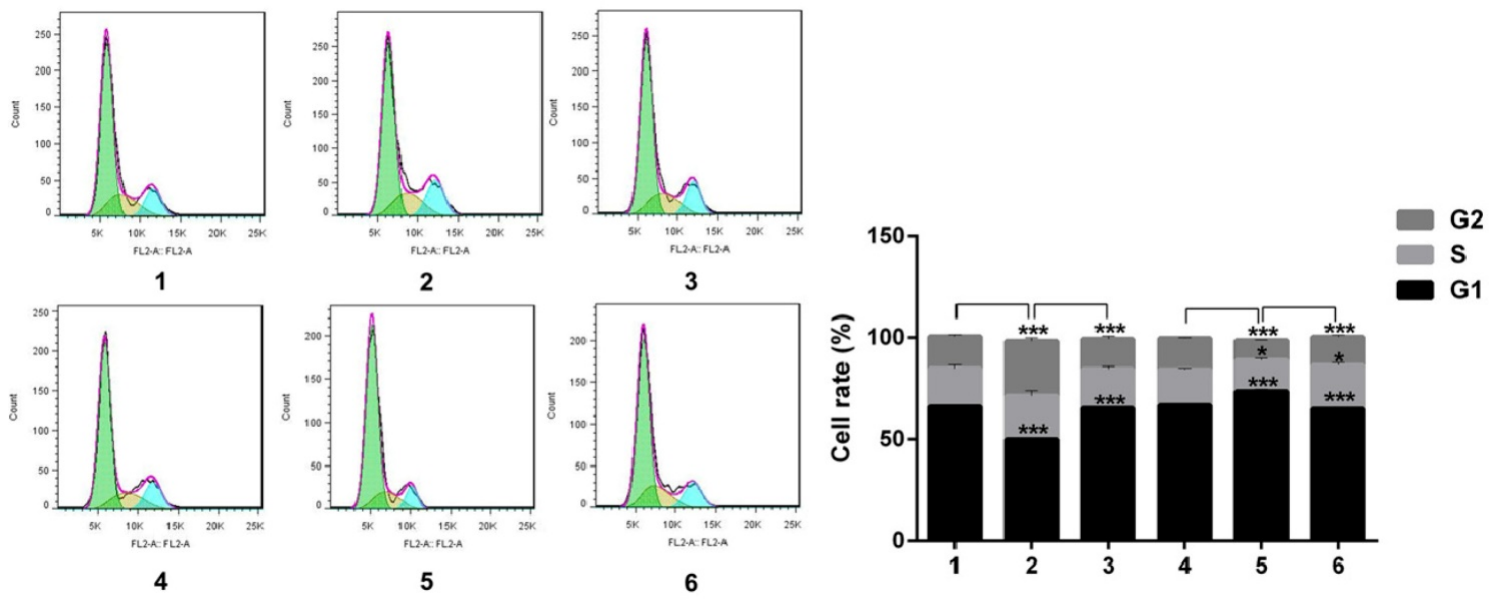
** Flow cytology analysis of cell cycle after Snail interference.** Notch1 increased the cells in phase S and G2, and decreased cells in phase G1 (group 1 vs group 2; group 4 vs group 5), the effect can be reversed by Snail (group 2 vs group 3; group 5 vs group 6). (1 = Snail NC + pCDNA3.1(+) + Cisplatin, 2 = Snail NC + pCDNA3.1(+)-NICD + Cisplatin, 3 = Snail siRNA+pCDNA3.1(+)-NICD + Cisplatin; 4 = pCDNA3.1(+) + Notch1 NC + Cisplatin; 5 = pCDNA3.1(+) + Notch1 siRNA + Cisplatin; 6 = pCDNA3.1(+)-Snail + Notch1 siRNA+ Cisplatin)

Notch1 increased the cells in phase S and G2, and decreased cells in phase G1 (group 1 vs group 2, p<0.001; group 4 vs group 5, p<0.001), the effect can be reversed by Snail (group 2 vs group 3, p<0.001; group 5 vs group 6, p<0.001). All the figure we provided represented only one of the multiple experiments (*P≤0.05, **P≤0.01, ***P < 0.001.) (Figure 7).

#### Immunofluorescence analysis of cell proliferation under Snail inducement

**Figure 8:**
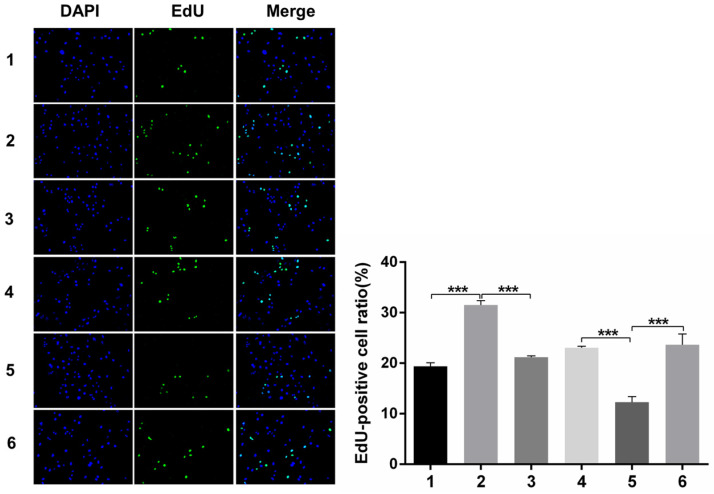
** Ki67 staining analysis of cell proliferation after Snail interference.** Over-expression of Notch1 promoted cell proliferation (group 1 vs group 2), while siRNA-mediated Notch1 knock-down inhibited cell proliferation (group 4 vs group 5), Snail would reverse the effect (group 2 vs group 3; group 5 vs group 6). Nuclear DNA was labelled in blue with DAPI. (1 = Snail NC + pCDNA3.1(+) + Cisplatin, 2 = Snail NC + pCDNA3.1(+)-NICD + Cisplatin, 3 = Snail siRNA+pCDNA3.1(+)-NICD + Cisplatin; 4 = pCDNA3.1(+) + Notch1 NC + Cisplatin; 5 = pCDNA3.1(+) + Notch1 siRNA + Cisplatin; 6 = pCDNA3.1(+)-Snail + Notch1 siRNA+ Cisplatin)

**Figure 9:**
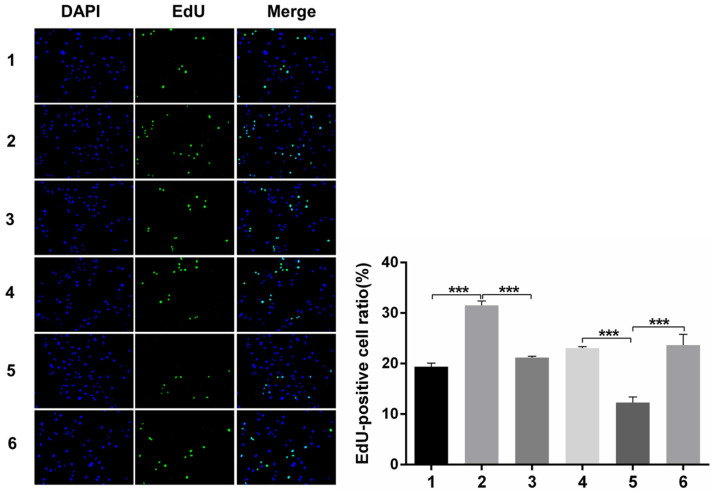
** Edu staining analysis of cell proliferation after Snail interference.** Over-expression of Notch1 promoted cell proliferation (group 1 vs group 2), while siRNA-mediated Notch1 knock-down decreased cell proliferation (group 4 vs group 5), Snail would reverse the effect (group 2 vs group 3; group 5 vs group 6). (1 = Snail NC + pCDNA3.1(+) + Cisplatin, 2 = Snail NC + pCDNA3.1(+)-NICD + Cisplatin, 3 = Snail siRNA + pCDNA3.1(+) -NICD + Cisplatin; 4 = pCDNA3.1(+) + Notch1 NC + Cisplatin; 5 = pCDNA3.1(+) + Notch1 siRNA + Cisplatin; 6 = pCDNA3.1(+)-Snail + Notch1 siRNA+ Cisplatin)

Nuclear DNA was labelled in blue with DAPI. Ki67 and Edu staining was marked in red. Ki67 and Edu staining showed over-expression of Notch1 promoting cell proliferation (group 1 vs group 2, p<0.001), while siRNA-mediated Notch1 knock-down decreasing cell proliferation (group 4 vs group 5, p<0.001), Snail would reverse the effect (group 2 vs group 3, p<0.001; group 5 vs group 6, p<0.001). All the figures we provided represented only one of the multiple experiments (*P≤0.05, **P≤0.01, ***P < 0.001.) (Figure 8, Figure 9).

## Discussion

There were three main findings of this study. Firstly, ovarian cancer tissue showed an increased protein expression of Notch1, Snail, MMP-2, N-cadherin and Vimentin, but had decreased expression of E-cadherin. Secondly, At the SKOV3 cell level, Notch1 was found to regulate the EMT process, promoting cell migration and invasion. Thirdly, again at the cellular level, Notch1mediated acquired chemo-resistance via facilitating EMT. Over-expression of Notch1 can promote EMT, inhibiting the apoptosis, and increasing the cells in phase S and G2, promoting cell proliferation.

Notch1 pathway, firstly reported by Thomas Hunt Morgan, is a highly conservative signaling pathway 11. A lot of studies have provided data on the role of the Notch1 in the development of tumor. Alniaimi AN reported that increased Notch1 expression is associated with poor overall survival in patients with ovarian cancer 12. The previous studies, which explored the close relationship between Notch1 and its role on ovarian cancer, were controversial. On one hand, a lot of literature confirmed the promoting role of Notch1 in pancreatic cancer, breast cancer, prostate cancer and ovarian cancer 13. Zou J et al. reported that Notch1 is required for hypoxia-induced proliferation, invasion and chemoresistance of T-cell acute lymphoblastic leukemia cells 14. Lian W et al. reported that AP-2α reverses vincristine-induced multi-drug resistance of SGC7901 gastric cancer cells by inhibiting the Notch pathway 15. Wang M et al. reported that pretreatment with the γ-secretase inhibitor DAPT sensitizes drug-resistant ovarian cancer cells to cisplatin by down-regulation of Notch signaling 16. On the other hand, recent literature confirmed the inhibiting role of Notch1 in non-small cell lung cancer 17. In this study, we once again confirmed Notch1 might promote tumor progression through regulating EMT process, which was consistent with our previous experimental results 5. Chemo-resistance plays an extremely important part in the fatality rate associated with ovarian cancer. Insensitivity of chemotherapy is one of the most important causes of treatment failure. So far, effective methods to reverse chemotherapy resistance have not been proven. As we mentioned in the background, EMT plays an important role in tumor chemo-resistance 8,9. Our study is the first to reveal the mechanisms of chemo-resistance in EOC mediated by Notch1. This may provide a new target for the treatment of ovarian cancer.

In addition, we also reviewed literatures about the application of Notch1 inhibitors in solid tumors. Chen J et al. Reported that inhibition of Notch signaling blocked growth of glioblastoma cell lines and tumor neutrospheres 18. Mizuna M et al. reported that the gamma secretase inhibitor MRK-003 attenuated pancreatic cancer growth in preclinical models 19. Richter. S et al. reported a phase I study of the oral gamma secretase inhibitor R04929097 in combination with gemcitabine in patients with advanced solid tumors 20. Schott AF et al concluded preclinical and clinical studies of gamma secretase inhibitors with docetaxel on human breast tumors 21. Krop I et al. reported that complete response was observed in Phase I pharmacologic and pharmacodynamic study of the gamma secretase (Notch) inhibitor MK-0752 in adult patients with advanced solid tumors 22. However, there is limited report about the application of Notch1 inhibitor in ovarian cancer. Combined with our previous research, we came to this conclusion that it would be reasonable to hypothesize that Notch1 signaling inhibition may favor anti-tumor and it might provide a new target for EOC, our study provides laboratory evidence for the future use of Notch1 inhibitors in ovarian cancer.

There are also three main limitations in our study. Firstly, due to the limited cases and funding, the sample size is relatively small, and the implementation of western blot was carried out on mixed samples according to literature. Secondly, only a representative EOC cell line SKOV3 was selected for experiment. It will be absolutely much better to extend conditioned media from other cell lines. Thirdly, due to financial constraints, the efficacy of Notch1 inhibitor at the cell level has not been further verified. All of the above will be our future research direction.

## Conclusion

Notch1 affects EOC cells chemo-resistance by regulating EMT. This may provide a new target for the treatment of ovarian cancer.

## Abbreviations

EOC: epithelial ovarian cancer
EMT: Epithelial-mesenchymal transition
Edu: 5-ethynyl-2′-deoxyuridine
